# Immunotherapy in microsatellite instability metastatic colorectal cancer: Current status and future perspectives

**Published:** 2021-08-04

**Authors:** Rodrigo Motta, Santiago Cabezas-Camarero, Cesar Torres-Mattos, Alejandro Riquelme, Ana Calle, Alejandro Figueroa, Miguel J. Sotelo

**Affiliations:** ^1^Department of Medical Oncology, Centro Oncologico Aliada; Lima, Peru; ^2^Functional Unit of Health Technology, Instituto Nacional de Enfermedades Neoplasicas; Lima, Peru,; ^3^Department of Medical Oncology, Hospital Universitario Clínico San Carlos; Madrid, Spain; ^4^Department of Medical Oncology, Hospital Nacional Guillermo Almenara Irigoyen; Lima, Peru; ^5^Oncological Research Unit, Clínica San Gabriel, Lima, Peru; ^6^Department of Medical Oncology, Hospital Universitario Infanta Cristina; Madrid, Spain; ^7^Department of Medical Oncology, Hospital María Auxiliadora; Lima, Peru; ^8^Department of Medical Oncology, Hospital Nacional Edgardo Rebagliati Martins; Lima, Peru

**Keywords:** anti-PD1, colon cancer, colorectal cancer, Immunotherapy, microsatellite instability

## Abstract

**Background:**

Colorectal cancer (CRC) is one of the most frequent and deadly malignancies worldwide. This specific pathology is composed of various molecular entities, with distinct immunological phenotypes. In addition to KRAS, NRAS, and BRAF mutation status, other druggable alterations such as those in HER2, MET, NTRK, ALK, and ROS1 have been identified in recent years offering new therapeutic options for some patients with CRC.

**Aim:**

This review will focus on the molecular biology, immunological fingerprints, and current clinical evidence for the use of immunotherapy in patients with CRC.

**Relevance for patients:**

High microsatellite instability (MSI-H) and mutations in mismatch repair genes constitute a new molecular entity within CRC, which is characterized by a high mutational and neoantigen burden, frequent immune cell infiltration, and where immune checkpoint inhibitors have shown high response and survival rates compared to microsatellite stable (MSS) tumors. Indeed, the approval of pembrolizumab in MSI-H tumors was the first agnostic FDA approval in solid tumors. While monotherapy with anti-programmed cell death protein-1 agents achieves objective response rates (ORR) of around 30% and 1-year overall survival (OS) rates of 76%, anti-PD1, and anti-CTLA4 combinations achieve a 55% ORR and a 1-year OS rate of 85%. Several ongoing trials are evaluating the use of different immunotherapy combinations, both in the advanced and early settings and in MSI-h and MSS CRCs.

## 1. Introduction

According to GLOBOCAN, in 2018 colorectal cancer (CRC) was the third most common malignancy and second in mortality worldwide, accounting for 10.2% of all new cancer cases, and 9.2% of all cancer deaths. Due to its especially high incidence in countries with a high human development index, CRC incidence has been proposed as a surrogate of socioeconomic development [1]. The incidence of CRC in the past 30 years has been especially increasing among those <50 years-old and at the expense of an increment in rectal cancer [2]. Alcohol, tobacco, overweightness and obesity, processed meats, high-fat diets and lack of physical activity, and genetics are the main risk factors behind the development of CRC [3-5]. Several predictive biomarkers have emerged to aid in the therapeutic decision-making in metastatic CRC, such as *KRAS* (40%)*, NRAS* (5–10%), and *BRAF* (8–10%) mutation status, tumor sidedness, microsatellite instability (MSI), and other less common alterations such as *HER2 (*2%)*, MET* (2%)*, NTRK* (0.2-2.4%)*, ALK* (0.2–2.4%), and *ROS*1 (0.2–2.4%) [6].

The terminology regarding MSI is not homogeneous. MSI is commonly described as a hyper-mutable phenotype, resulting from a defective DNA mismatch repair (MMR) system, that leads to the presence of alternate sized repetitive DNA sequences that may (Lynch syndrome) or may not (sporadic cases) be present in the corresponding germline DNA [7]. Lynch syndrome accounts for 3-5% of cases of CRC, giving rise to MSI-high (MSI-h) CRC, characterized by a high mutational burden and high neoantigen exposition, which make them prone to respond to immune checkpoint inhibitors (ICIs) [8]. An additional 10-15% of CRC also harbor MSI-h while being sporadic and not inherited [9-11].

MSI has an incidence of up to 15–20% in local CRC and has clinical relevance in early stages. Stage III MSI tumors exhibit higher rates of lymphovascular invasion and perineural invasion. However, there is no clear association between MSI-h Stage III CRC and benefit from adjuvant chemotherapy. In addition, MSI-h is associated with poor response to chemotherapy in Stage II tumors. This may be due to the solid evidence indicating that MSI-h Stage II or III CRC are associated with fewer odds of relapse and death compared to microsatellite stable (MSS) CRC. Therefore, MSI-h status in Stage II or III CRC may allow to de-escalate adjuvant treatment in selected cases [12-15].

Considering the high incidence and mortality of CRC and the importance of immunotherapy in MSI-h CRC, as well as in other solid tumors, our aim was to thoroughly review the immunogenicity of MSS and MSI CRC, as well as the current evidence and future perspectives for the use of immunotherapy in this disease.

## 2. Colon Cancer as an Immunogenic Disease

CRC is one of the most studied neoplasms from a genetic perspective [6]. Progression from a benign adenoma to a malignant carcinoma occurs through a series of cumulative events, including chromosomal abnormalities, genetic mutations, and epigenetic changes. These changes lead to the inactivation of tumor suppressor genes, mutations in DNA repair genes, and the activation of oncogenes [5].

A microsatellite consists of repeating sequences of 1–6 nucleotides. The characteristic distribution is different every 15–65 nucleotides of satellite DNA, mainly located near the end of the chromosome [16,17]. The DNA MMR system is responsible for correcting errors in DNA replication. Mutations in MMR genes lead to the accumulation of mutations favoring malignant transformation [18]. Therefore, MSI-H tumors are associated with the production and accumulation of hundreds of somatic mutations, which lead to a high neoantigen exposure that favors the initiation of a robust antitumor immune response [7,8,18,19]. Three types of MSI can be distinguished: MSI-H, MSI-low (MSI-L) and MSS [10]. MSI-H gives rise to hypermutated tumors, a highly effective neoantigen presentation and infiltration by T-effector cells, as wells as immunosuppressive cells such as myeloid-derived suppressor cells (MDSCs) and T-regulatory (Tregs) cells, which explain the high response rates but also the emergence of resistance mechanisms observed with ICIs [6,9,10,19].

In the past decade, several attempts have been made to understand and classify CRC based on its molecular profile and in search of possible therapeutic targets [10,14]. The Cancer Genome Atlas classification (2013) groups patients into chromosomal instability (CIN) (84%), hypermutated (13%) and ultramutated (3%) profiles. The hypermutated group is characterized by dMMR, MSI, MLH1-sll, CIMP-h, BRAF-mut, and SCNA-low [20]. The CRC Subtyping Consortium (2015) described four consensus molecular subtypes (CMS) in CRC, each with different mutations and specific molecular-pathway activations that harbor different prognoses and therapeutic implications [11,18,19]. Immune subtype (CMS 1) accounts for 15–20% of all CRC. CMS 1 is MSI-H, hypermutated, harbors *BRAFV600E* mutations, shows a high hypermethylated phenotype (CIMP) more frequently than other subtypes, and predominates in right-sided CRC. In addition, it shows immune activation, with the presence of CD8+ and CD4+ T cells of the T-helper 1 (Th1) phenotype, M1 macrophages and expression of programmed cell death protein-1 (PD-1), programmed death ligand 1 (PD-L1), CTLA4, and LAG3. While the prognostic value of CMS subtypes depends on the disease setting and therapies that are used, CMS1 is associated with a poor prognosis in those with *KRAS, BRAF mutant*, and sporadic and distal tumors [18,19]. Notably, MSI-H tumors have a higher angiogenic potential and higher microvascular density that can facilitate a broad, local, and inflammatory response. This may explain why patients with CMS1 obtained a greater survival benefit from bevacizumab compared to cetuximab in CALGB-80405 [21]. Furthermore, infiltration by tumor-infiltrating lymphocytes (TILs) is associated with a better prognosis in tumors with MSI [6,8,9,18,19,22]. The mesenchymal molecular subtype (CMS 4) represents up to 25% of CRC cases. It is MSS and shows CIN, TGF-β upregulation, *KRAS* and *PIK3CA* mutations, and IGBP3 overexpression. It harbors a poor prognosis due to the development of a dense stroma with immunosuppressive cell infiltration by Tregs, MDSCs, M2 macrophages, and few CD8+ T cells. The Canonical subtype (CMS 2) accounts for 35-40% of cases of CRC. It is characterized by MSS, CIN, WNT/Myc pathway activation and *EGFR* dependence, which may explain why patients with CMS2 achieved a longer survival with cetuximab within CALGB-80405. CMS2 shows an “immune-desert” phenotype and predominates in left-sided CRC. Finally, the metabolic subtype (CMS 3), which represents 13% of CRC, is characterized by MSI and CIN, metabolic dysregulation with matrix remodeling and angiogenesis, and is enriched in *KRAS* mutations. It predominates in right-sided CRC and shows an immune-mixed phenotype enriched in cells expressing PD-1, Th17 cells, and naïve T/B cells [10,18].

## 3. Biomarkers Used in the Treatment of CRC

Around 75% of advanced CRC harbor predictive biomarkers that allow choosing the best therapeutic option. *KRAS* mutations occur in 40% of CRC, with a up to 85–90% of them, occurring in exon 2, codons 12 and 13, and the remainder in exons 3 and 4 [11,23]. For decades, KRAS mutations have been considered “undruggable.” However, sotorasib a KRAS-G12C mutation inhibitor has been recently approved by the FDA for KRAS-G12C-mutant non-small cell lung cancer (NSCLC), after demonstrating high response and survival rates [24]. Although, KRAS-G12C is a rare mutation in mCRC, this results in NSCLC provide a prove of concept that targeting KRAS may also be feasible in other entities, such as mCRC [25]. *NRAS* mutations occur in 5-10% of patients, in exons 2, 3, and 4. B*RAF* mutations develop in 8–10% of cases, the majority (90%) in the *V600E locus*, meanwhile *BRAF V600E* mutations account for 90% of BRAF mutations in CRC, with a 10-fold more activity compared to the wild-type counterpart [11]. KRAS, NRAS, and BRAF mutations constitutively activate the RAS/BRAF/ERK pathway and therefore render upstream inhibition by anti-EGFR agents useless [6,18]. The different embryological origin of the left and right-sided CRC, explain their different biology and clinical behavior. Indeed, RAS wild-type right-sided CRC respond less to anti-EGFR agents compared to left-sided CRC. Prospective trials have consistently shown the favorable impact in survival rates with anti-EGFR agents in patients with RAS and BRAF wild-type left-sided metastatic CRC. On the other hand, anti-VEGF agents are the biological agent of choice in RAS-mutant or right-sided mCRC [26-33]. Other druggable alterations occur in smaller subsets of patients, such as *HER2* and *MET* amplifications, *NTRK1-3*, *ALK*, and *ROS1* fusions, with several small studies demonstrating the activity of anti-HER2 agents, MET inhibitors and NTRK, ROS1, and ALK inhibitors in CRC, and other cancer types [6,18].

As already mentioned, MSI, which accounts for 5-7% mCRC, has prognostic and therapeutic implications [19]. MSI is associated with high mutational and neoantigen burdens that permit tumor identification by the immune system, thus explaining the high responses and survival rates observed with immunotherapy in MSI-H CRC [7-11,33-39]. There are at least two mechanisms by which tumors acquire MSI: Lynch-syndrome and Lynch-like syndrome associate with inherited mutated genes (accounting for 5–6% of CRC). Sporadic cases (accounting for 8–10% of CRC) are secondary to the MLH1-promoter hypermethylation which is more common in BRAF-mutant tumors. There are two methods commonly used in the diagnosis of MSI: Immunohistochemistry (IHC) and polymerase chain reaction (PCR) [6,9-11,41-43]. Next generation sequencing (NGS), a third alternative method, which is everyday more accessible, has shown high concordance rates with the former methods [10,44]. Most IHC MSI tests consist of a four-antibody panel that includes MLH1, PMS2, MSH2, and MSH6 [10]. NGS and IHC are high sensibility and specificity methods. NGS has a sensitivity of 95.8% and specificity of 99.4%, with a positive predictive value of 94.5%, and negative predictive value of 99.2% as compared to PCR. For IHC, sensitivity ranges from 80.8% to 100.0%, and specificity from 80.5% to 91.9% [45].

However, reported response rates to anti-PD1 are variable and often <50% in patients with MSI-H, suggesting that additional predictive biomarkers are needed. Recent studies point out that tumor mutational burden (TMB) appears to be an important predictive biomarker of response to immunotherapy in multiple tumor types independent of MSI status or PD-L1 expression. TMB-high tumors are thought to harbor an increased neoantigen burden, making them immunogenic, and responsive to immunotherapy. Studies have shown that TMB in MSI-H mCRC is generally elevated, but still quite variable. Although still a few and small-sized studies have addressed this issue, TMB-high mCRC shows a strong association with higher objective response and longer progression-free survival (PFS) with immunotherapy in comparison with TMB-low mCRC, marking TMB a potential predictive biomarker in this population [46-48].

Increasing evidence demonstrates that the cancer evolution is strongly dependent on the complex tumor microenvironment in which it develops. Although not standardized yet, the immunoscore (IS) is a direct measure of T-cell infiltration into tumors, based on the amount of lymphocyte populations, especially CD3 and CD8-positive T cells, commonly found in high amounts in MSI-H or dMMR patients. The IS provides a scoring system ranging from low to high, helping to predict and stratify patients who could benefit from immunotherapy [49]. In addition, up to 20% of MSS CRC harbor a similar profile to MSI-H tumors. Among these, a small percentage of MSS CRC (<1%) are secondary to POL-E and POL-D mutations, characterized by an ultra-mutator phenotype, and have also been shown to respond to PD-1 inhibitors [6,9-11,42,43]. Is well known that there is an important association between lifestyle factors and the risk of developing CRC, especially dietary factors. Human body is colonized by more than 100 trillion microbes most of which are bacteria, of eukaryotic and archaeal species, many of which are in the gut. Gut microbiota colonization starts at birth and is remodeled according to diet, lifestyle, disease, aging, drug consumption, and other environmental factors. The gut of a healthy individual is mainly composed of a specific type and number of microbes that help to regulate homeostasis, inflammation, metabolism and immunity. The role of gut microbiota in such mechanisms is a relatively new research field, but as an immuno and metabolic modulator, could potentially affect the efficacy of immunotherapy in cancer patients [50-52]. Further prospective studies are needed to validate IS, POL-E and POL-D mutations and microbiota as predictive factors.

## 4. Action Mechanism of ICIs

### 4.1. PD-1/PD-L1 inhibitors

PD-1/PD-L1 is expressed by B and T cells, macrophages, and dendritic cells. Their expression leads to a weakened host immune response and a consequent poor prognosis [7,39]. PD-1 is a cell surface inhibitory receptor expressed on activated T cells, pro-B cells, and macrophages. PD-L1 is the ligand for PD-1 and is commonly expressed by tumor cells, but also by immune suppressor cells such as Tregs, M2 macrophages, and MDSCs. Under physiological conditions, the binding of PD-L1 to the receptor PD-1 produces a negative feedback that prevents the host from being attacked by its own immune system [7,39]. However, malignant cells take advantage of this mechanism through the expression of PD-L1 to evade immunosurveillance. Binding PD-1 to PD-L1 is known to disable the T-lymphocytes effector function by decreasing their activation and proliferation. PD-1 (nivolumab or pembrolizumab) and PD-L1 (durvalumab, atezolizumab, or avelumab) inhibitors block the PD-1/PD-L1 inhibitory interaction, thus allowing cytotoxic T-lymphocytes to exert - their anti-tumor effect, particularly in tumors with a high neoantigen burden and an already established T-cell-mediated immune response. As discussed below, this explains why PD-1 inhibitors have shown high response and survival rates in MSI-H CRC [8,46].

### 4.2. Anti-CTLA4 agents

CTLA-4 is a B7/CD28 family member that inhibits T cell function and is constitutively expressed by Tregs, but can also by upregulated by CD4+ T cells. CTLA-4 mediates immunosuppression by competing with the co-stimulatory receptor CD28 in the binding to their ligands CD80 and CD86 during antigen presentation. Therefore, under physiological conditions CTLA-4 inhibits the activation of T cells, favoring tolerance to self-antigens. Tumors favor CTLA-4 signaling, thus preventing antigen presentation and specific T-cell activation. Anti-CTLA4 agents such as ipilimumab and tremelimumab have shown activity in different cancers, including MSI-H CRC, demonstrating a synergistic, or at least an additive effect when combined with anti-PD(L)1 agents. However, compared to PD(L)1 inhibitors, anti-CTLA4 agents account for a higher rate of grade >3 immune-related adverse events (AEs) [8,46].

## 5. Current Evidence for the Use of Immunotherapy in CRC

The first immunotherapy used in CRC was the antihelmintic levamisole in the 1980s due to its known immunomodulatory effects, although it was soon abandoned after 5-FU and leucovorin became the standard chemotherapy backbone [10].

With the advent of modern immunotherapy, initial studies in CRC sought to evaluate the effect of anti-PD-1 agents in the second- or third-line settings. The most relevant trials with ICIs in this setting will be discussed below and are summarized in Table 1.

**Table 1:** Most relevant studies with immune checkpoint inhibitors in the second-line setting of advanced colorectal cancer

Trial	Phase	Agent (Dosage)	N	Target population	ORR	PFS	OS	G3-4 TRAEs
Keynote-028	Ib	Pembro (10 mg/kg/q2wk)	23	PD-L1+ mCRC	1/23	-	-	-
Keynote-028	II	Pembro (10 mg/kg/q2wk)	78	MMRd cancers	CRC: 52% Non-CRC: 54%	NR 2-y PFS: 53%	NR 2-y OS: 64%
Keynote-164	II	Pembro (200 mg/q3wk)	124	MSI-H/MMRd mCRC A: 1 prior line B: >2 prior lines	A, B: 33%	A: 2.3 m B: 4.1 m	A: 31.4 m B: NR	A: 16% B: 13%
CheckMate-142	II	Nivo (3 mg/kg/q2wk)	74	MSI-H/MMRd mCRC with 1 prior line	31%	1-y PFS: 50.4%	1-y OS: 73.4%	-
CheckMate-142	II	Nivo (3 mg/kg/q2wk) + Ipi (1 mg/kg/q4wk) × 4 cycles →Maintenance Nivo	119	MSI-H/MMRd mCRC with >1 prior lines	55%	1-y PFS: 71%	1-y OS:85%	32%
IMblaze 370	III	Atezo (840 mg/q2wk) + Cobimetinib (60 mg/qd 1–21) versus atezo (1200 mg/q3wk) versus regorafenib (160 mg/qd 1-21)	363	MSS mCRC (95%) MSI-H mCRC (5%)	-	-	8.87 versus 7.10 vs 8.51 m	-

Atezo: Atezolizumab, Ipi: Ipilimumab, mCRC: Metastatic colorectal cancer, MMRd: Mismatch-repair deficient, MSI-H: Microsatellite instability-high, MSS: Microsatellite stable, N: Number of patients, Nivo: Nivolumab, NR: Not reached, ORR: Objective response rate, OS: Overall survival, Pembro: Pembrolizumab, PFS: Progression-free survival, TRAEs: Treatment-related adverse events

### 5.1. Second- or further-line immunotherapy in advanced CRC

The phase Ib trial Keynote-028 evaluated the safety and preliminary efficacy of pembrolizumab 10 mg/kg for up to 2 years or until disease progression or unacceptable toxicity in a cohort of 23 patients with advanced CRC among 137 patients with PD-L1+ solid tumors. After a median follow-up of 5.3 months, 65% of the patients had progressed and the sole CRC responder was MSI-H. Most common AEs were fatigue (13%), stomatitis (9%), and asthenia (9%) [30].

Le *et a*l. [7] hypothesized that tumors with high mutational and neoantigen burdens due to mismatch-repair defects (MMRd) could benefit from ICIs, and thus designed a phase 2 trial to evaluate the activity of pembrolizumab 10 mg/kg/q2wk in patients with MMR-deficient (MMRd) mCRC, MMR-proficient (MMRp) mCRC, and MMRd non-CRCs. Among 11 patients with MMRd mCRC, immune-related (IR) objective response rates (ORR) and 20-week PFS achieved 40% and 78%, respectively, versus 0% and 11% among 18 patients with MMRp CRC. While median PFS and overall survival (OS) were 2.2 and 5.0 months in MMRp mCRC, they were not reached in MMRd mCRC, demonstrating a HR for death of 0.22 (*P*=0.05), and a HR for disease progression of 0.10 (*P*<0.001). Among the 9 patients with MMRd non-CRCs, IR ORR and 20-week PFS reached 71% and 67%, respectively. Interestingly MMRd tumors harbored a mean of 1782 mutations compared to a mean of 73 mutations in MMRp tumors (*P*=0.007).

Le *et a*l. [38], in an update of the study after a median follow-up of 12.5 months, reported results from 78 patients with 12 different MMRd tumor types, among which 32 had confirmed germline MMRd tumors, while seven additional cases had a family history consistent with Lynch syndrome. ORR achieved 53% and disease control rate (DCR) 77%, with an average time to any response of 21 weeks, and to complete response of 42 weeks. ORR in CRC and non-CRC were similar (52% vs. 54%), as well as between Lynch syndrome-associated and non-Lynch syndrome-associated cancers (46% vs. 59%, *P*=0.27). While median PFS and OS were not reached, estimates of PFS and OS at 1 year were 64% and 76%, and 53% and 64% at 2 years, respectively. Among the 11 and seven patients achieving complete and partial responses, respectively, and taken off therapy after 2 years of pembrolizumab, no tumor progressions had been observed at 8.3 and 7.6 months of follow-up post-therapy. The authors did not find differences in TMB between responders and non-responders. Interestingly, after identifying seven neoantigen-reactive T Cell Receptors (TCRs) present at very low titers in peripheral blood (below 0.02%) they observed how 4 of them increased rapidly after pembrolizumab and then descended just before radiologic response.

In the phase II trial KEYNOTE-164, Le *et a*l. [38] evaluated the safety and activity of pembrolizumab 200 mg q3wk in 124 patients with MSI-H/MMRd CRC treated with ≥2 prior lines of standard therapy (cohort A; N=61) or with ≥1 prior lines of therapy (cohort B; N=63). ORR, the primary endpoint, was 33% in both cohorts with median duration of response (DOR) not reached in either of them. After a median follow-up of 31.3 months in cohort A and 24.2 months in cohort B, median PFS and OS were 2.3 and 31.4 months in cohort A, and 4.1 and not reached in cohort B. Toxicity was manageable with no new safety signs compared to previously reported trials. Grade 3-4 treatment-related AEs (TRAEs) were 16% in cohort A and 13% in cohort B.

In 2017, Overmann *et a*l. [39], in Checkmate-142, a multi-cohort phase II trial, evaluated the activity and safety of nivolumab 3 mg/kg q2wk among 74 patients with MSI-H/MMRd mCRC that had progressed to ≥1 lines of therapy. ORR was 31.1% and 12-week DCR achieved 68.9%. After a median follow-up of 12 months, median DOR had not been reached and 1-year PFS and OS achieved 50.4% and 73.4%, respectively. There were no differences in ORR or 12-wk DCR depending on PD-L1 expression or clinical history of Lynch syndrome.

In 2018, Overmann *et al*. [40] published data from the nivolumab plus ipilimumab cohort of Checkmate-142. Efficacy and safety of the mentioned combination (Nivolumab 1 mg/kg plus ipilimumab 3 mg/kg q3wk for four doses followed by nivolumab 3 mg/kg q2wk) were evaluated in 119 patients with MSI-H/MMRd mCRC progressing to ≥ 1 lines of systemic therapy. ORR and 12-wk DCR were 55% and 80%, respectively. Median DOR was not reached and 1-year PFS and OS achieved 71% and 85%, respectively. Notably, treatment was associated with statistically significant improvements in symptoms, role functioning, and quality-of-life. Grade 3–4 TRAEs occurred in 32% with no new safety signs compared to other cancer types.

Finally, IMblaze 370 was an open-label, phase 3 trial that randomized 363 patients in a 2:1:1 method to receive atezolizumab (840 mg IV q2wk) plus cobimetinib (60 mg orally qd days 1-21 in a 28-day cycle), atezolizumab monotherapy (1200 mg IV q3wk), or regorafenib (160 mg orally qd days 1–21 every 28 days). Notably, the MSI-H patient number was limited to 5% of enrolled patients. There were no differences in median OS among the three arms of the study (8.87 vs. 7.10 vs. 8.51 months), and therefore the trial did not meet its primary endpoint in the unselected mCRC population [40].

Other studies are evaluating de role of ICIs either alone or in combination with other ICIs or with cytotoxic chemotherapy and targeted therapies in the second-line setting (Table 2).

**Table 2:** Most relevant ongoing trials with immune checkpoint inhibitors in advanced and early-stage colorectal cancer

Trial	Phase	Agent (Dosage)	N	Target population	Primary endpoint
NCT03104439	II	Nivo + Ipi + Radiotherapy	80	Second-line MSS mCRC	DFS
NCT03608046	II	Avelumab + Cetuximab + CPT-11	59	Second-line MSS mCRC	PFS, OS
NCT03800602	II	Nivo + Metformin	24	Second-line MSS mCRC	PFS, OS
NCT03832621	II	Nivo + Ipi + Temozolomide	27	Second-line MSS mCRC	ORR, PFS, OS
NCT03007407	II	Durva + anti-CTLA4 (Tremelimumab)	33	Second-line MSS mCRC	ORR, Safety
NCT03642067	II	Nivo + anti-LAG3 (relatlimab)	96	Second-line MSS mCRC	ORR, Safety
POLE-M	III	5-FU-based chemo +/- sequential avelumab	-	Resected stage III CRC with MSI-H/MMRd /POLE-Mutant	DFS
ATOMIC	II	5-FU-based chemo +/- atezo→atezo × 6 months	-	Resected stage III CRC with MSI-H/MMRd	DFS OS

AEs: Adverse events, Atezo: Atezolizumab, CRC: Colorectal cancer, CPT-11: Irinotecan, Durva: Durvalumab, Ipi: Ipilimumab, mCRC: Metastatic colorectal cancer, MMRd: Mismatch-repair deficient, MSI-H: Microsatellite instability-high, MSS: Microsatellite stable, N: Number of patients, Nivo: Nivolumab, Pembro: Pembrolizumab

### 5.2. Immunotherapy in the first line of advanced CRC

A number of trials have studied the activity of ICIs in the first-line setting of biomarker-unselected mCRC [9]. However, as shown in the second-line setting, ICIs did not meet the primary endpoint in these trials since the majority of patients had MSS mCRC. This was the case of the MODUL trial that combined the anti-PD-L1 atezolizumab with chemotherapy and bevacizumab [8,9,14].

On the other hand, a number of trials have demonstrated the efficacy and safety of ICIs in the first-line setting in MSI-H/MMRd mCRC (Table 3).

**Table 3:** Most relevant studies with immune checkpoint inhibitors in the first-line setting of advanced colorectal cancer

Trial	Phase	Agent (Dosage)	N	Target population	ORR	PFS	OS	G3-4 TRAEs
MODUL	-	Atezo	-	mCRC	-	-	-	-
CheckMate-142	II	Nivo (3 mg/kg/q2wk) + Ipi (1 mg/kg/q4wk) x 4 cycles →Maintenance Nivo	45	MSI-H/MMRd mCRC chemo-naïve	60%	1-y PFS: 77%	1-y OS: 83%	20%
Keynote-177	III	Pembro (200 mg/q3wk) versus 5FU-based chemo + beva or cetu	307	MSI-H/MMRd mCRC chemo-naïve	43.8% versus 33.1%	16.5 versus 8.2 m (HR 0.60)	NR	22% versus 66%

Atezo: Atezolizumab, Ipi: Ipilimumab, mCRC: Metastatic colorectal cancer, MMRd: Mismatch-repair deficient, MSI-H: Microsatellite instability-high, N: Number of patients, Nivo: Nivolumab, NR: Not reached, ORR: Objective response rate, OS: Overall survival, Pembro: Pembrolizumab, PFS: Progression-free survival, TRAEs: Treatment-related adverse events

In another cohort of Checkmate-142, nivolumab plus low-dose ipilimumab (Nivolumab 3 mg/kg plus ipilimumab 1 mg/kg q3wk for four doses followed by nivolumab 3 mg/kg q2wk) was evaluated in 45 patients with MSI-H/MMRd mCRC. ORR and DCR were 60% and 84%, and after a median follow-up of 19.9 months, 1-year PFS and OS were 77% and 83%, respectively. Grade 3-4 TRAEs occurred in 20%, thereby causing 11% of the patients to discontinue treatment. These results, presented as ASCO GI 2020, demonstrate a robust and lasting clinical benefit, as well as a manageable safety profile, thus establishing this combination as a potential new standard in this setting, especially in patients with high tumor burden in need of a tumor response [34].

Keynote-177 was a phase 3, open-label trial that randomized 307 patients with MSI-H/MMRd mCRC in the first-line setting to receive pembrolizumab 200 mg q3wk or 5-FU-based chemotherapy with or without bevacizumab or cetuximab q2wk. Cross over to pembrolizumab was allowed in the chemotherapy arm after disease progression. After a median follow-up of 32.4 months, PFS was longer for pembrolizumab compared to chemotherapy (16.5 vs. 8.2 m; HR, 0.60; 95% CI 0.45–0.80; *P*=0.0002). OS results were still immature. ORR reached 43.8% in the pembrolizumab group compared to 33.1% in the chemotherapy arm. Eighty-three percent of responses were still ongoing at 2 years in the pembrolizumab group compared to 35% in the chemotherapy group. Toxicity was also favorable to pembrolizumab with a 22% TRAEs rate compared to 66% in the chemotherapy group. Interestingly, all subgroups benefited from pembrolizumab in terms of PFS. However, the KRAS/NRAS-mutant subgroup achieved less benefit, a result that merits confirmation in further clinical trials [35].

While OS results are still pending, and considering the potential cross-over effect (at the time of cutoff, 59% of patients in the chemotherapy arm had crossed over to anti-PD(L)1 agents either within or outside the trial), this is a practice-changing trial and its results probably establish pembrolizumab as a new first-line standard in MSH-I/MMRd CRC [35].

## 6. FDA-Approved Indications and Patient-centered Recommendations of Use

Results from Keynote-028 and the two cohorts from CheckMate-142, led to the FDA approval of pembrolizumab (May 2017), nivolumab (August 2017), and nivolumab combined with low-dose ipilimumab (July 2018) in the second-line setting for MSI-H/MMRd mCRC [9]. Recently, the EMA has also approved these agents in MSI-H/MMRd mCRC. While there are no trials comparing pembrolizumab with nivolumab or with nivolumab/ipilimumab, efficacy seems comparable between both anti-PD1 agents in terms of ORR, DOR, PFS, and OS. While the nivolumab/ipilimumab combination accounts for a higher ORR (55%) than nivolumab (ORR: 31%) or pembrolizumab (ORR: 32%) monotherapies, the anti-PD1/anti-CTLA4 combination is also more toxic, almost doubling the rate of Grade 3-4 TRAEs. Therefore, as other authors, we suggest to use the nivolumab/ipilimumab combination in patients with a favorable ECOG PS (0–1), a high tumor burden, or a rapidly evolving tumor where a high ORR is a priority. On the other hand, in cases of low-tumor burden or in fragile patients due to age, comorbidities or an ECOG PS 2, it may be more prudent to use single anti-PD-1 agents, given their better safety profile compared to the anti-PD1/anti-CTLA4 combination.

**Figure 1:**
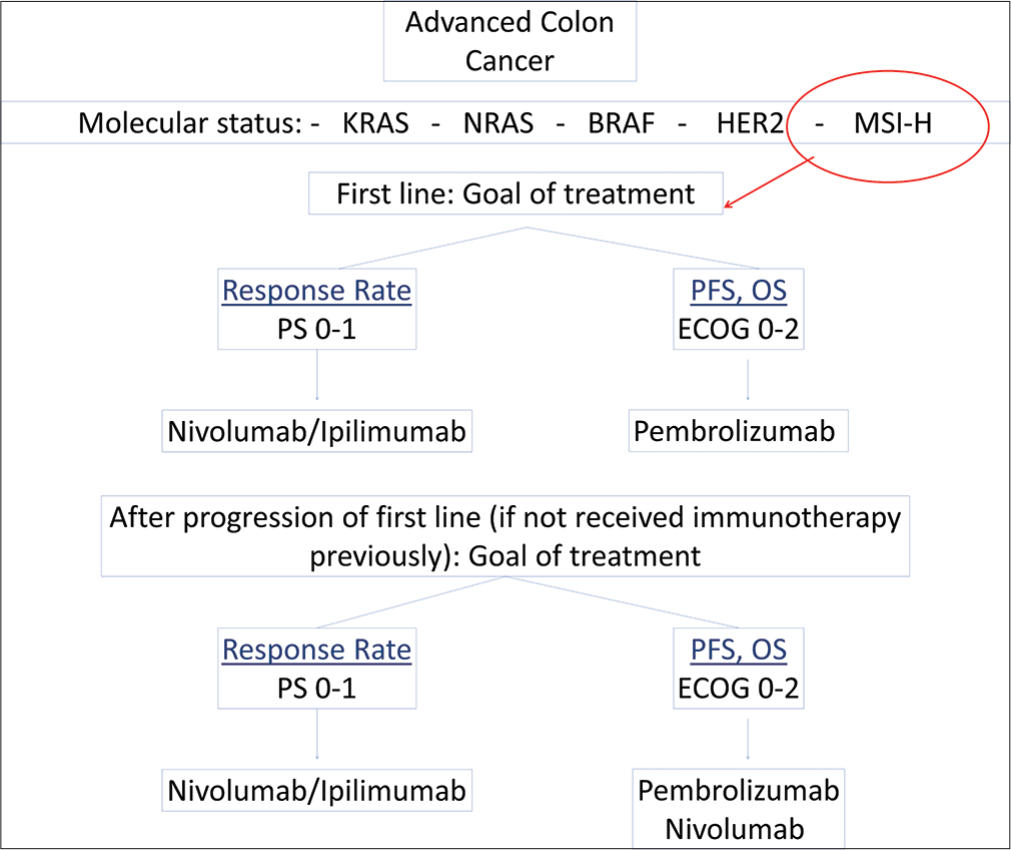
Treatment algorithm of advanced colorectal cancer with microsatellite instability-high

Although only a number of trials have evaluated the efficacy and safety of anti-PD1 agents in the first-line setting, it must be noted that in Keynote-177, median PFS with pembrolizumab doubled that of chemotherapy (16.5 m vs. 8.2 m), while ORR achieved 43.1% with pembrolizumab (vs. 31% with chemotherapy) [35]. The FDA (June 2020) and EMA (January 2021) approved pembrolizumab for first-line treatment of MSI-H/dMMR CRC. In CheckMate-142, the nivolumab/ipilimumab combination achieved a 60% ORR but caused 11% of the patients to discontinue therapy due to toxicity [35]. Therefore, regarding the choice of anti-PD1 monotherapy or anti-PD1/anti-CTLA4 combination, we believe that the same recommendations given in the second-line setting, apply in the first-line setting (Figure 1). As in the subgroup analysis of Keynote-177, patients with the *KRAS/NRAS* mutation derived no benefit from pembrolizumab. This result is worth evaluating in specific prospective trials. Finally, it must be noted that OS results in Keynote-177 were still immature at the time of publication, and we should therefore wait for the final OS analysis, as well as results from ongoing trials to emit a definitive recommendation [36].

### 6.1. Advanced colorectal cancer

KRAS, NRAS, BRAF, HER2, and MSI must be evaluated in all patients with advanced colon cancer. If the tumor shows high MSI (MSI-H), immunotherapy is the treatment of choice. If the aim of therapy is to achieve a high response rate and the patient has a PS 0-1, has no relevant comorbidities and is not an elderly patient (>70 years-old), the combination of nivolumab/ipilimumab should be considered. If the patient has a low tumor burden and therefore a high response rate is less relevant, or is the patient has limitating comorbidities or has an ECOG PS 2, anti-PD1 monotherapy should be the treatment of choice. The same considerations apply both in the first- and second-line setting regarding the choice of immunotherapy in MSI-H/MMRd mCRC. mCRC: metastatic colorectal cancer, MMRd: mismatch-repair deficient, MSI: microsatellite instability, MSI-H: MSI-high.

## 7. Immunotherapy Resistance in CRC

Although immunotherapy has demonstrated being a new option of therapy, markers of resistance to immunotherapy have been discovered in patients with solid tumors. Deletions or mutations in JAK1/2, IFNGR1/2, and IRF1118 have been reported, particularly JAK1 and JAK2. Mutations that inactivate JAK1 or JAK2 lead to both acquired as well as primary resistance to anti-PD1 therapy. Truncating mutations in B2M lead to impaired MHC Class I antigen presentation and generation of immune escape variants that fail to elicit a T cell response. Stability of chromatin remodeling complexes (PBRM1, ARID2, and BRD7) in tumors contributes to immunotherapy resistance, which inabilities the recruitment of MMR genes during DNA repair and subsequently diminish the neoantigen load. Furthermore, immunoediting has been reported as a mechanism for resistance. Immunoediting suggests that constant interactions between the immune system and cancer cells result in selection of subclones within the tumor that lack expression of neoantigens, conferring poor immunogenicity and resistance to immunotherapy. Mechanisms of resistance for immunotherapy in CRC is still unclear and further studies must be done to acknowledge this topic [53,54].

## 8. Future Perspectives

The field of immunotherapy is growing rapidly with recent approvals in the MSI-H population. However, many questions remain unresolved and the majority of patients with CRC, which are MSS, do not benefit from currently approved immunotherapies. As a consequence, there are multiple ongoing studies that are assessing the role of different immunotherapy modalities in new scenarios.

### 8.1. Metastatic setting

#### 8.1.1. PD(L)1 inhibitors combined with other agents in MSS mCRC

Regorafenib is a multi-tyrosine kinase inhibitor that blocks many pathways, including CSF1R, whose inhibition may reduce the recruitment of immune-suppressive tumor-associated macrophages to the tumor microenvironment. A phase I/IB trial evaluated the combination of nivolumab and regorafenib in refractory pMMR mCRC, where 28 patients received nivolumab 240 mg IV q2wk and regorafenib according to a dose escalation design. The combination was considered safe. Dose modifications (reductions or interruptions) for drug-related AEs occurred in four patients. mPFS was 5.7 months and mOS was not reached after a median follow-up of 4.6 months. REGONIVO, a phase Ib trial, evaluated dose-limiting toxicity during the first 4 weeks of treatment with the same regimen in 50 pretreated patients (25 mCRC and 26 metastatic gastric cancer [mGC]) to estimate the maximum tolerated dose. One patient had MSI while the rest had MSS/MMR-p tumors. The combination of regorafenib/nivolumab had a manageable safety profile and encouraging antitumor activity. The most common Grade 3 treatment-related AEs were rash (12%), proteinuria (12%), and palmar-plantar erythrodysesthesia (10%). ORR was 40%, and mPFS achieved 5.6 months and 7.9 months in patients with mGC and mCRC, respectively. The BACCI trial was a phase II study, with, unfortunately negative results. This study randomized in a 2:1 ratio, 133 patients with unselected mCRC (86% MSS/MMRp) to receive capecitabine-bevacizumab combined with atezolizumab or placebo. In MSS-only patients, HR for PFS was 0.67 (0.44–1.03) and 1-year OS achieved 52% in the atezolizumab arm (compared to 43% in the control group) [9,55,56].

Cobimetinib inhibits MEK1/MEK2 in the MAPK pathway and has been shown to alter the tumor environment and T-cell responses to promote anti-tumor immune activity. IMblaze370, a phase III trial, evaluated survival with atezolizumab/cobimetinib regimen versus atezolizumab versus regorafenib in MSS/MSI-L heavily pretreated mCRC, finding negative results. Most patients (91.7%) were MSS or MSI-L, and 54.3% had RAS mutations. There were not statistically significant OS differences between the three groups. mOS was 8.9 months with atezolizumab/cobimetinib, 7.1 months with atezolizumab, and 8.5 months with regorafenib. There were no differences in PFS either, with a HR of 1.25 for atezolizumab/cobimetinib versus regorafenib and HR of 1.39 for atezolizumab versus regorafenib [57].

The CCTG CO.26 study, investigated somatic variants contributing to plasma TMB (pTMB) in MSS patients using cell-free DNA analysis performed with the GuardantOMNITM test. Patients with pTMB >28 mutations/megabase (21% of them with MSS tumors) had the greatest OS benefit for durvalumab and tremelimumab (HR 0.34, 90% CI, 0.18–0.63, *P*=0.070) with a worse OS in the best supportive care arm (HR 2.59, 90% CI, 1.46–4.62). Of 4044 mutations detected, 67.2% were subclonal and after removing them from pTMB calculation, median pTMB decreased 5.8 mutations/megabase. However, this clonal pTMB remained predictive of durvalumab and tremelimumab improving OS (HR 0.19, 90% CI 0.08–0.45, p = 0.039) with pTMB >10.6 mutations/megabase (14.1% pts). This study suggests that subclonal and clonal mutations could have a predictive value for immunotherapy in MSS mCRC patients [58].

The COMMIT trial is a phase III, open-label study where MSI-H/MMRd chemo-naïve mCRC patients will be randomized to 3 arms: atezolizumab + FOLFOX/Bevacizumab, an atezolizumab monotherapy arm, and a control arm with FOLFOX/Bevacizumab. The primary endpoint is PFS (NCT02997228) [9].

#### 8.1.2. Bispecific antibodies

The use of T cell-targeted bispecific antibodies is a growing area of research. T cell-dependent bispecific antibody-induced T cell activation, which can eliminate tumor cells independent of MHC engagement, is expected to be a novel breakthrough immunotherapy against refractory cancer. *In vitro* and *ex vivo* data suggest that a prolonged presence of the drug in target tissues may result in significant T-cell recruitment, activation and expansion to/in target tissues, potentially resulting in substantial anti-tumor activity. The carcinoembryonic antigen (CEA)-TCB bispecific antibody simultaneously binds to CD3 expressed on T cells and the CEA, thus promoting T cell proliferation and cytokine release, and subsequently “warming-up” the tumor microenvironment [59].

The CEA-TCB antibody in combination with atezolizumab was shown to increase tumor inflammation and response in a Phase I trial in patients with mCRC [8,60]. Ongoing trials are studying the pharmacokinetics, safety and tolerability of bispecific monoclonal antibodies such as MT110, MGD019, or ZW25; alone or with chemotherapy/immunotherapy in mCRC, mainly in heavily pretreated patients, as well as in other solid neoplasms (NCT00895323, NCT03866239, NCT00635596, NCT03761017, and NCT03929666).

#### 8.1.3. Vaccines and intra-tumor immunotherapies

While results from vaccination therapy in mCRC have been discouraging, a number of trials are currently ongoing [8-10,14].

Intra-tumor immunotherapies are an area or active research in mCRC. The TLR9 agonist lefitolimod did not show efficacy as a maintenance therapy in the IMPALA phase 3 trial. At present, a trial (NCT03256344) is testing the combination of systemic atezolizumab with the intratumoral administration of talimogene laherparepvec, an attenuated and genetically-modified herpes simplex virus type I that is approved for the treatment of advanced melanoma [61].

#### 8.1.4. Adoptive cell therapy (ACT)

In a case report, Tran *et al*. [62] described the case of a 50-year-old patient with mCRC while enrolled in a trial of ACT using intravenously administered *ex vivo* expanded TILs (NCT01174121). This patient’s TILs showed a polyclonal CD8+ T-cell response against mutant KRAS G12D. After a nonmyeloablative lymphodepleting chemotherapy consisting of cyclophosphamide and fludarabine, the patient received a single infusion of the *ex vivo* expanded KRAS G12D-reactive TILs, followed by five doses of interleukin-2. An objective regression of the seven pulmonary metastases was observed. When one of the lesions progressed, study of the surgical specimen revealed a loss of chromosome 6 encoding the HLA-C class II MHC molecule, thereby demonstrating the alteration in the antigen presentation machinery as the mechanism behind the observed acquired resistance.

Two studies evaluated the safety and clinical activity of FOLFOX administered concurrently with NKG2D CAR T-cells. The SHRINK study (NCT03310008) evaluated the autologous NKG2D CAR product CYAD-01 while the alloSHRINK study (NCT03692429) evaluated an allogeneic analog of CYAD-01. The concurrent administration of FOLFOX aims to improve the likelihood of clinical responses in solid tumors by favoring T cell infiltration into the immunosuppressive TME, improving engraftment of CAR T-cells due to the lymphodepletion induced by chemotherapy, and likely increasing the NKG2D-ligand expression in tumor tissues targeted by the NKG2D CARs. In the context of the CYAD-101 allogenic administration, while the graft-versus-host disease (GvHD) effect is controlled by the inhibition of TCR signaling in the study product, the administration of chemotherapy (FOLFOX) will also contribute to control GvHD reactions through elimination of the previously adoptively-transferred CYAD-101 cells [63]. CAR-T immunotherapy is another type of ACT that, although only approved in some hematologic malignancies, is also being tested in solid tumors, including CRC (NCT03152435) [9-11,64].

### 8.2. Adjuvant setting

In the MSI-H/MMRd population there are currently several ongoing studies in the adjuvant setting. In patients with Stage III CRC, the ATOMIC trial is evaluating adjuvant therapy with FOLFOX with or without atezolizumab (NCT02912559). The POLEM study is comparing fluoropyrimidine-based chemotherapy with or without the anti-PDL1 avelumab in Stage III MMRd or POLE-mutant CRC (NCT03827044). An interesting study is evaluating adjuvant pembrolizumab versus placebo in patients with resected MSI-H/MMRd CRC that show persistent circulating tumor DNA after surgery. At least two trials are evaluating the role of vaccines in the adjuvant setting: One with autologous dentritic cells (NCT02415699) and the other with the AVX701 vaccine consisting of an alpha virus encoding the CEA protein (NCT01890213) [11].

### 8.3. Neoadjuvant setting

The introduction of immunotherapy in the neoadjuvant setting of CRC is preceded by the growing number of studies with promising results in other entities such as melanoma and NSCLC. Neoadjuvant immunotherapy may allow for a more effective immune response (more efficient antigen presentation to T cells that might be less exhausted than in more advanced stages), and also may improve surgical outcomes due to the potential tumor reduction. It may also allow for a potent and durable “vaccine” effect with the development of a specific immune response against tumor antigens [9-11,65].

The NICHE trial evaluated the safety and activity of nivolumab plus ipilimumab in 21 MMRd CRC and the same combination with celecoxib in 20 MMRp CRC. In the MMRd group, the major pathological response rate (with <10% viable tumor cells) was 95%. However, the rate of Grade 3-4 TRAEs was 13%, which may be problematic in a pre-surgical setting [11].

In locally advanced rectal cancer, the VOLTAGE-A trial evaluated nivolumab 240 mg q2wk for 5 cycles pre-surgery after capecitabine-based chemoradiation. The trial demonstrated a complete pathological response rate of 30% in MSS CRC and of 60% in MSI-H CRC. In the same setting, another trial is evaluating the anti-PD1 dostarlimab followed by chemoradiation (NCT04165772) [11].

## 9. Conclusions

CRC is a heterogenous disease with an increasingly well-known immunological basis. While a majority of tumors will not respond to current PD-1/PD-L1 targeted immunotherapies, up to 20% will harbor MSI, a demonstrated marker of benefit from ICIs that has led to FDA-approval of anti-PD1 agents (pembrolizumab, nivolumab) or anti-PD1/anti-CTLA4 combinations (nivolumab plus ipilimumab) in metastatic CRC. Given the different rates of benefit and toxicity between anti-PD1 monotherapies and the anti-PD1/anti-CTLA4 combination, it is suggested to individualize their use depending on the performance status of the patient and the need for a high response rate. Current data point to a clear impact in response rate and survival when ICIs are used in the first-line setting, and have shown promising activity in the neoadjuvant setting. Results from currently ongoing and future trials with ICIs and other immunotherapy modalities in the (neo) adjuvant and advanced settings, both for MSI-H/MMRd and MSS CRC, constitute a rapidly expanding area of investigation and are eagerly awaited.

## Conflicts of Interest

The authors declare no conflicts of interest.
